# Occurrence of Hepatitis C Virus infection in type 2 diabetic patients attending Plateau state specialist hospital Jos Nigeria

**DOI:** 10.1186/1743-422X-6-98

**Published:** 2009-07-08

**Authors:** James A Ndako, Georgebest O Echeonwu, Nathaniel N Shidali, Iliyasu A Bichi, Grace A Paul, Ema Onovoh, Lilian A Okeke

**Affiliations:** 1 Department of Virology, Federal College of Veterinary and Medical Laboratory Technology, Vom, Nigeria; 2 Department of Medical Laboratory Sciences, University of Maiduguri, Nigeria

## Abstract

**Background:**

Glucose intolerance is observed more in patients with HCV infection compared with control subjects with liver disease, Initial studies suggested that Hepatitis C virus infection may be an additional risk factor for the development of diabetes mellitus. This study was therefore carried out to determine the correlation of HCV infection and diabetes.

**Methods:**

Three hundred (300) confirmed type 2 diabetic patients were screened for hepatitis C virus antibodies at the Plateau state specialist hospital, Jos, using Grand diagnostic test strip. Questionnaire comprising of age, sex, family history on diabetes, duration of disease and marital status were issued to subjects.

**Results:**

Overall result showed that the prevalence rate of HCV infection was 33(11%). In response to diabetic status, females subjects had a higher prevalence of 178(59.3%) compared to males 122(40.7%). Those aged 47–57 recorded the highest seroprevalence 10(30.3%) to the Hepatitis C Virus, while Patients without family history of diabetes showed a higher seroprevalence of 13(39.4%). Subjects who never had any blood transfusion recorded a prevalence rate of 6(18.2%). Marital status showed no significant difference [(P = 0.275; P.0.05)]. Considering duration of developing diabetes, patients within the range of 1–10 years diabetic status recorded the highest prevalence rate 25(75.8%) compared to other ranges considered.

**Conclusion:**

This study hence, suggests a relatively strong association between HCV infection and diabetes, this therefore call for an urgent approach strategy in the control and management of this disease of the endocrine system.

## Background

Various epidemiological studies have suggested that hepatitis C virus (HCV) infection is a risk factor for the development of diabetes mellitus (DM) type 2. The etiological factors were initially thought to be cirrhosis but further studies differentiating between HCV and hepatitis B virus (HBV) related infection have shown that patient with HCV infection have a higher prevalence of Diabetes mellitus type-2 [1].

### Pathogenesis

Most persons who become infected with HCV viraemia persist, accompanied by variable degrees of hepatic inflammation and fibrosis. Earlier studies of chronic HCV infection suggests that only a small number of hepatocytes become infected, but more recent studies suggest that 50% or more harbor the virus[2]. HCV is most efficiently transmitted through transfusion of infected blood, transplantation of infected organs, and sharing injection drug equipment [3].

### Diagnosis

The diagnosis of HCV infection can be made by detecting either anti-HCV or HCV RNAof anti-HCV is recommended for routine testing of asymptomatic persons and should include use of both enzyme immunoassay (EIA) and supplemental or confirmatory testing with an additional, more specific assay. Use of supplemental antibody testing (i.e., RIBA) for all positive anti-HCV results of EIA is preferred, particularly in settings where clinical services are not provided directly [4].

### Aim

Type 2 diabetes is a debilitating disease condition especially in people above 30 years of age and this may evolve throughout their life-span. Hence, the co-infection of type 2 diabetes and HCV has been established to worsen these condition, with this scenario it has become very necessary for a screening exercise to determine the prevalence rate of HCV among diabetic patients so as to increase awareness of the populace and health practitioners on the dangers of the co-infectious state of this virus with diabetes.

## Materials and methods

Our study was carried out according to the ethical standards for human experimentation. After explaining the aim of the study and the possible need for blood tests, written informed consent were obtained and ethical clearance granted.

### Subjects

The subjects included in this research project were 300 known type 2 diabetic patients attending the hospital for check-ups.

### Study Location

The study was carried out at the plateau state specialist hospital, Jos South local government area, Plateau state.

### Sample Collection

3 ml of blood was collected from each patient by vene- puncture using sterile syringes and needles into a sterile container with a screw cap. The blood was allowed to clot, and the sera samples were dispensed into a dry clean cryovial and stored at -20°c prior use.

### Method of Assay

The grand diagnostic rapid test kit was used to analyze the samples for HCV antibodies. This is a rapid chromatographic immunoassay for the qualitative detection of antibody to HCV in serum.

#### Questionnaire

Structured questionnaire were administered to the subjects, which covers clinical characteristics, demographic characteristics and social behaviors, which could be possible risk factors to acquiring HCV.

### Statistical Analysis

General descriptive analysis was used to analyze the responses from the questionnaire and results were expressed as percentages. The chi square test was used to compare categorical data at 95% confidence interval, odds ratio and significance level were taken at P ≥ 0.05.

## Results

Seroprevalence of HCV among type 2 diabetes patients was found to be 11% (33 out of 300 type 2 diabetes patients were positive for anti-HCV antibody).

The age group of 47–57 years had a prevalence rate of 3.3%, followed by those aged 69 years and above with 3.0%, while subjects aged 36–46 years recorded 2.3%, in contrast individuals aged 25–35 years recorded 0%. These data showed a significant difference within the age groups as P > 0.05 with a chi-square value of 34.803. example (table 1).

**Table 1:** Age distribution of HCV in type 2 diabetic patients.

Age group		Negative	Positive	Total
**25–35**	Count	51	0	51
	% within hcv status	19.1%	0%	17.0%
	% Total	17.0%	0%	17.0%
**36–46**	Count	95	7	102
	% within hcv status	35.6%	21.2%	34.0%
	% Total	31.7%	2.3%	34.0%
**47–57**	Count	73	10	83
	% within hcv status	27.3%	30.3%	27.7%
	% Total	24.3%	3.3%	27.7%
**58–68**	Count	38	7	45
	% within hcv status	14.2%	21.2%	15.0%
	% Total	12.7%	2.3%	15.0%
**69 above**	Count	10	9	19
	% within hcv status	3.7%	27.3%	6.3%
	% Total	3.3%	3.0%	6.3%
**Total**	Count	267	33	300
	% within hcv status	100.0%	100.0%	100.0%
	% Total	89.0%	11.0%	100.0%

Pearson Chi-square value = 34.803 P value = .000 i.e. P < 0.05

The females had 6.3% of positivity within while the males recorded 4.7. Despite the fact that females had a higher prevalence rate, statistically P > 0.05, it is hence not a significant value, example (table 2).

**Table 2:** Distribution of HCV pattern based on the sex of individual screened.

Sex		Negative	Positive	Total
Female	Count	159	19	178
	% within hcv status	59.6%	57.6%	59.3%
	% Total	53.0%	6.3%	59.3%
Male	Count	108	14	122
	% within hcv status	40.4%	42.4%	40.7%
	% Total	36.0%	4.7%	40.7%
Total	Count	267	33	300
	% within hcv status	100.0%	100.0%	100.0%
	% Total	89.0%	11.0%	100.0%

Pearson Chi-square value = 0.047

P value = 0.828 i.e. P > 0.05

Considering individuals with family history of diabetes, it was realized that that those without family history had a higher prevalence of 6.7% while those with family history recorded a prevalence of 4.3%. Using the chi-square test it was found that Pearson chi square value is 46.829 with a significant P value of (P < 0.05), example (table 3).

**Table 3:** Association of HCV among subjects with family history of diabetes.

FHOD		Negative	Positive	Total
No	Count	255	20	275
	% within hcv status	95.5%	60.6%	91.7%
	% Total	85.5%	6.7%	91.7%
Yes	Count	12	13	25
	% within hcv status	4.5%	39.4%	8.3%
	% Total	4.0%	4.3%	8.3%
Total	Count	267	33	300
	% within hcv status	100.0%	100.0%	100.0%
	% Total	89.0%	11.0%	100.0%

FHOD = family history of diabetes.

Pearson Chi-square value = 46.829

P value = .000 i.e. P < 0.05

Blood transfusion history was also compared in HCV status in the type 2 diabetes patients. Those that never had any blood transfusion showed a higher prevalence rate of 9.0% while those with an evidence of blood transfusion recorded 2.0%, showing a chi-square value of 15.223 and P < 0.05, example (table 4).

**Table 4:** Association of HCV with blood transfusion amongst subjects screened.

Blood transfusion		Negative	Positive	Total
No	Count	259	27	286
	% within hcv status	97.0%	81.8%	95.3%
	% Total	86.3%	9.0%	95.3%
Yes	Count	8	6	14
	% within hcv status	3.0%	18.2%	4.7%
	% Total	2.7%	2.0%	4.7%
Total	Count	267	33	300
	% within hcv status	100.0%	100.0%	100.0%
	% Total	89.0%	11.0%	100.0%

Pearson chi-square value = 15.223

P value = .000 i.e. P < 0.05

When marital status comprising those divorced, married, and single individuals was closely compared among those with HCV status a prevalence of 0%, 10.0%, and 0.3% were obtained respectively with no significant difference recorded, example (table 5).

**Table 5:** Association of Marital status with HCV in type 2 diabetes patients.

Marital status		Negative	Positive	Total
Divorce	Count	12	0	12
	% within hcv status	4.5%	.0%	4.0%
	% Total	4.0%	.0%	4.0%
Married	Count	235	32	267
	% within hcv status	88.0%	97.0%	89.0%
	% Total	78.3%	10.7%	89.0%
Single	Count	20	1	21
	% within hcv status	7.5%	3.0%	7.0%
	% Total	6.7%	.3%	7.0%
Total	Count	267	33	300
	% within hcv status	100.0%	100.0%	100.0%
	% Total	89.0%	11.0%	100.0%

Pearson Chi_square value = 2.582

P value = 0.275 i.e. P > 0.05

Considering the duration of diabetes condition among subjects recruited, in this study, participants were grouped into: 1–10 years, 11–15 years, and 16 years above. Those with 1–10 years of Diabetic condition showed a higher prevalence of 8.3%, followed by 11–15 years with 2.7%, while subjects 16 years above recorded 0%, example (table 6).

**Table 6:** Association of HCV with duration of type 2 diabetes amongst the patients screened.

Years range		Negative	Positive	Total
1–10	Count	256	25	281
	% within hcv status	95.9%	75.8%	93.7%
	% Total	85.4%	8.3%	93.7%
11–15	Count	8	8	16
	% within hcv status	3.0%	24.2%	5.3%
	% Total	2.7%	2.7%	5.3%
16 above	Count	3	0	3
	% within hcv status	1.1%	.0%	1.0%
	% Total	1.0%	.0%	1.0%
Total	Count	267	33	300
	% within hcv status	100.0%	100.0%	100.0%
	% Total	89.0%	11.0%	100.0%

Pearson chi-square value = 41.380

P value = 0.00 i.e. P < 0.05

## Discussion

The prevalence rate of 11% recorded in this study is in agreement with the work of [5] who also recorded 11.5% against 2.5% when prevalence of HCV infection was checked among diabetic patients and blood donors respectively.

It was noted that patients who have been infected with HCV might have acquired diabetes due to damage to β-cells of the pancreas so as to induce diabetes. Where as those that have diabetes before being infected with HCV might suffer from insulin resistance resulting in more adverse effects of diabetes than those with HCV infection alone.

It was observed from this study that the occurrence of HCV among type 2 diabetes was higher in subjects aged 47–57 years with 3.3%, This agrees with the findings of Mehta et. al. [1,6] who showed that individuals of >40 years are more prone to type 2 diabetes. Equaly in this study, subjects less than 36 years of age recorded a very low prevalence of HCV infection of 0%, which also agrees with the report of Mehta *et al*. [1,7] that type 2 diabetes occurs more often with HCV infection in those older than 40 years of age. From the statistical analysis P value is 0.000 which denotes P < 0.05 which is statistically significant.

The distribution of HCV infection in males and females were found to be 4.2% and 6.3% respectively, despite this fact, the statistical analysis gave a P value of 0.828 i.e. P < .05 which is not significant. Hence, it can be concluded that the distribution of HCV infection in both sexes is the same.

When HCV status was analyzed against previous history of blood transfusion between those that have received and those that had never received blood transfusion, there was a significant difference with P value = 0.000 in which those that consented to have had transfusion recorded a lower prevalence of 2.0% while those without prior history of blood transfusion recorded 2.7%. This scenario suggests that infection with HCV in this group might not be as a result of blood transfusion. This finding agrees with the work of Simo *et al*. [5] were he did not observe any significant difference among subjects with previous blood transfusion to the HCV infection with 21.8% as against 16.7% in intravenous drug users, while 10.2% against 5.5% prevalence was observed between blood donors and diabetes patients with CV infection respectively.

A significant difference was observed in participants with a family history of diabetes mellitus and those without, with a P value of 0.000. Interestingly, this coincides with the work of Muller *et al *[8] and Del *et al *[9] Where it was found that increased occurrence was associated with family history of diabetes mellitus and this variable may be one of the reasons of higher frequency of diabetes mellitus type 2 in this group of patients. This study also shows that the prevalence of HCV infection in type 2 diabetes patients within the status had no significant difference with P value = 0.275 were P > 0.05. This might suggest less risk of sexual transmission of HCV infection in this category of subjects.

In relation to the duration of type 2 diabetes onset, the analysis showed that those who suffered from diabetes within the period of 10 years had a seroprevalence of 8.3% as compared with those who had suffered from diabetes for 16 years and above with 0%. This reflects the long term damage of the virus in the liver resulting to low sugar metabolism such organ due to hepatocyte damage. [10,11].

## Conclusion

There is a significant association between hepatitis C virus and type 2 diabetes from the findings of this research work which might be on the increase, this calls for an urgent need to educate the populace on the dangers of the co-infection of HCV and diabetes it's also vital for all health care practioners to come to terms with the early diagnosis and Management of this condition in affected patients. However more prospective studies is recommended to include individuals at greater risk for both HCV infection and type 2 Diabetes to enable a firm relationship between these 2 conditions to be better established.

## Competing interests

The authors declare that they have no competing interests.

## Authors' contributions

JA conceived of the study and carried out the study design, and drafting of the manuscript. IB carried out the immunoassays, participated in data collection and Statistical analysis. GE Participated in drafting of the study design. NS Participated in study coordination. GP Participated in Data collection. EO Contributed in data analysis. LA Participated in the immunoassays. All authors read and approved the final manuscript.
